# Exploring resting-state functional connectivity invariants across the lifespan in healthy people by means of a recently proposed graph theoretical model

**DOI:** 10.1371/journal.pone.0206567

**Published:** 2018-11-08

**Authors:** Paolo Finotelli, Ottavia Dipasquale, Isa Costantini, Alessia Pini, Francesca Baglio, Giuseppe Baselli, Paolo Dulio, Mara Cercignani

**Affiliations:** 1 Department of Mathematics, Politecnico di Milano, Milan, Italy; 2 MRI Lab, IRCCS, Fondazione Don Carlo Gnocchi, Milan, Italy; 3 Department of Electronics, Information and Bioengineering, Politecnico di Milano, Milan, Italy; 4 Neuroimaging Laboratory, Santa Lucia Foundation, Rome, Italy; South-Central University for Nationalities, CHINA

## Abstract

In this paper we investigate the changes in the functional connectivity intensity, and some related properties, in healthy people, across the life span and at resting state. For the explicit computation of the functional connectivity we exploit a recently proposed model, that bases not only on the correlations data provided by the acquisition equipment, but also on different parameters, such as the anatomical distances between nodes and their degrees. The leading purpose of the paper is to show that the proposed approach is able to recover the main aspects of resting state condition known from the available literature, as well as to suggest new insights, perspectives and speculations from a neurobiological point of view. Our study involves 133 subjects, both males and females of different ages, with no evidence of neurological diseases or systemic disorders. First, we show how the model applies to the sample, where the subjects are grouped into 28 different groups (14 of males and 14 of females), according to their age. This leads to the construction of two graphs (one for males and one for females), that can be realistically interpreted as representative of the neural network during the resting state. Second, following the idea that the brain network is better understood by focusing on specific nodes having a kind of centrality, we refine the two output graphs by introducing a new metric that favours the selection of nodes having higher degrees. As a third step, we extensively comment and discuss the obtained results. In particular, it is remarkable that, despite a great overlapping exists between the outcomes concerning males and females, some intriguing differences appear. This motivates a deeper local investigation, which represents the fourth part of the paper, carried out through a thorough statistical analysis. As a result, we are enabled to support that, for two special age groups, a few links contribute in differentiating the behaviour of males and females. In addition, we performed an average-based comparison between the proposed model and the traditional statistical correlation-based approach, then discussing and commenting the main outlined discrepancies.

## Introduction

### Functional magnetic resonance in neuroscience

Undoubtedly, the functional magnetic resonance imaging (fMRI) constitutes a fundamental technique to examine brain function by using blood oxygen level–dependent (BOLD) contrast. Ever since it was demonstrated that fMRI is sensitive to spontaneous brain activity at rest (i.e., in the absence of a task), the so-called resting state fMRI (rsfMRI or R-fMRI) has gained great popularity for studying brain connectivity.

What makes fMRI so appealing for neuroscientists is that it can be applied in minimally-compliant populations such as young children, adolescents, adults, elderly as well as in people with neurodegenerative diseases or mental disorders.

While BOLD contrast has been used for nearly 3 decades to localize the neuronal activity associated with a specific task or stimulus, it is now established that, even at rest, the BOLD signal exhibits low-frequency spontaneous fluctuations. These oscillations are characterized by temporal correlations across spatially distinct brain regions and are thus believed to reflect the degree of “functional connectivity” (FC) (see for example [1]). The explanation for this baseline activity in the absence of external stimuli is that the brain maintains some sort of “stand-by” condition, which allows the activity to be resumed very quickly on demand [2]. Alternative explanations speculate that resting activity is devoted to prime the brain to respond to future stimuli, or to maintain relationships between areas that often work together to perform tasks. It may even consolidate memories or information absorbed during normal activity (see for example [3]).

Regardless of the specific function of resting-state activity, it forms a substantial part of neural physiology, as indicated by the amount of energy devoted to it: blood flow to the brain during rest is typically just 5–10% lower than during task-based experiments (see [4]). These patterns of synchronous activities can be measured using rsfMRI, and have been shown to be reproducible and associated with specific functional networks [5].

### Default mode network

Among those functional networks, the Default Mode Network (DMN) has received most attention. The term “default mode” was originally introduced by Raichle et al. in 2001 [6] to describe resting brain function (see also [7]). Other resting-state networks have been characterized by a high level of reproducibility, and functional disconnection has been implicated in several neurological and psychiatric disorders [8]. The field has slowly moved towards an increasingly abstract description of functional connections across the whole brain, based on a graph theory approach (see for example [9], [10], [11]), where any network can be described by means of its nodes, or vertices (in the case of the brain these would be grey matter areas), and the connections (also called edges, or links) between them.

The DMN is most active when the brain is at rest, while it deactivates when the brain is directed outwards or engaged in a cognitive task [12], [13], [14]. For this reason, the DMN is believed to be associated with introspection and general cognition. All the areas involved in the DMN are associated with high-level functions, such as memory (medial temporal lobe and precuneus), the theory of mind, or the ability to recognize others as having thoughts and feelings similar to one's own (medial prefrontal cortex); and integration of internal thoughts (posterior cingulate).

The strength of correlation between the nodes’ activity at rest can be used to weight the edges, and a number of topological parameters can be computed to characterize the network in terms of efficiency, modularity, segregation and integration.

### The threshold problem

While graph theory is a powerful approach for investigating changes occurring to the brain, several challenges and pitfalls remain to be addressed. Many steps are required to compute a graph from rsfMRI data, and some of them are still partially controversial, in particular, those that impose arbitrary choices, all of which can substantially impact on the results. One of the most critical points concerns the definition of a “threshold” for accepting a connection as “real” as opposed to noise. There are several approaches for defining thresholds. Maybe the most popular one, when data are originated by fMRI experiments, is correlation-based, meaning that a particular value $\tilde{r}$, between 0 and 1, is selected. Hence, only the links characterized by a correlation index greater than $\tilde{r}$ are considered. This method, though very common, could be a bit crude, since defining connectivity purely based on the correlation between functional time-series neglects the contribution of the underlying anatomical structure. For instance, a node *v* of high degree (i.e. the number of connections involving *v*) is automatically removed from the analysis in case all the edges whose *v* is an endpoint have correlation index below the threshold. On the other hand, a great number of connections could reveal to be significant from a neurobiological point of view, so that cutting-off *v* could cause the loss of important information on the brain behaviour. As it is conceivable that functional connectivity expresses structural connectivity, the edges of the graph should be weighted accordingly.

### Aging of the functional network

There is a great interest in describing and understanding the changes occurring in functional networks with age and gender, as a consequence of the hypothesised link between connectivity and function of the brain. Using a range of complementary approaches, several research groups have shown dramatic changes occurring during the lifespan. By means of a multivariate technique, Ferreira et al. [15] showed that normal aging is not only characterized by decreased resting-state FC within the DMN, but also by ubiquitous increases in internetwork positive correlations and focal internetwork losses of anticorrelations (involving mainly connections between the DMN and the attentional networks). Their outcomes seem to reinforce the notion that the aging brain undergoes dedifferentiation processes with loss of functional diversity. Using graph theory, Xu et al. [16] found that the whole-brain network is more easily fully connected in young adulthood than that in late adulthood, indicating that the central regions, frontal lobe, parietal lobe, and limbic lobe possibly occupy more resources in late adulthood. In addition, they pointed out a selective loss of connectivity strength within the temporal lobe and occipital lobe in late adulthood. Other changes occurring with aging include reduced small-worldness and connectivity density. FC studies based on neurophysiological methods (see for example [17]), also suggest the occurrence of age-related alterations of functional resting-state connectivity. Among the regions whose FC is more directly associated with cognitive performance are the posterior cingulate/precuneus and medial temporal lobe. The interaction between age and gender has also been explored, suggesting a faster cross-sectional FC decline with age in females [18]. Globally, the results of this study indicate that although both male and female brains show small-world network characteristics, male brains were more segregated and female brains were more integrated. These sex differences in rsfMRI are more likely to reflect sexual dimorphisms in the brain rather than transitory activating effects of sex hormones [19]. One important consequence of these findings is that age and sex should be controlled for in FC studies of young adults.

### Our work

A main goal of the paper is to identify the strongest functional connections that are different in males and females, through the lifespan and at resting state. In order to address this issue, we apply a modified model of FC that has been recently proposed [20]. In this model (in the following referred to as FD model, where F stands for “functional” and D stands for “distance”) the computation of the connectivity between each pair of nodes is not only based on the strength of the statistical correlations between different cerebral areas (measured by the correlation coefficient r), but also on the physical distance between the nodes, and their degree in the corresponding graph. The choices of functional and structural data can be done according to different criteria and methods (e.g. [21], [22], [23], [24]), without affecting the application of the FD model.

Also, as an alternative approach to the thresholding problem, we apply a 2-step method previously proposed, and already applied to real fMRI data [23]. This procedure aims in selecting a first threshold from the analysis of the histogram of the available data provided by the acquisition system, and then applies a second threshold on the matrices provided by the FD model. Consequently, the relevant links are selected from a suitable neighbour of interest, and, in the meantime, fulfil the request of having high weight according to the FD model. This reflects in that nodes having higher degrees have a higher probability of being preserved in the resulting network, which, in our opinion, could be preferable in view of understanding the areas playing a central role during the brain activity. Without pretending to solve definitively the thresholding issue, our proposal should be intended as an alternative operative approach to provide meaningful weights to the brain connections, based on the various kinds of involved connectivity and typical graph parameters and metrics.

Furthermore, we performed a model comparison analysis whose aim is to compare the FD model with the traditional statistical correlation-based approach, from now on referred to as *pure functional connectivity* (pFC) approach. This analysis, based on the corresponding sample-averaged representative matrices, outlined a few connections that, though neglected by the pFC, seem to be important at resting state, and suggest a deeper investigation in possible future works.

The paper is organized as follows.

First, the FD model and the 2-step thresholding procedure have been briefly summarized, also focusing on their explicit application to real data. Our study involved 133 subjects, both males and females of different age, with no evidence of neurological diseases or systemic disorders. We have shown how the model applies to the sample, where the subjects have been grouped into 28 different groups (14 of males and 14 of females), according to their age. From the obtained intensities we derived two graphs (one for males and one for females), that can be realistically interpreted as representative of the neural network during the resting state. As a result, we get a great overlapping between the outcomes concerning males and females. Then, following the idea that the brain activity can be better understood by focusing on specific nodes having a kind of centrality, we have refined the two previous graphs by introducing a new metric that favour nodes having higher degrees. This led to identify the strongest links in each one of the considered groups, showing that, both for males and for females, a main role seems to be played by the precuneus, the cingulate gyrus, the frontal pole, the paracingulate gyrus, and the occipital cortex. These are characterized both by high functional strength and degree and are consistent with the DMN as previously described in the literature (see for instance [6], [7], [12] and [25]).

Despite this, some intriguing differences appeared, which motivated a deeper local investigation, based on the statistical analysis of the results. As a consequence, we were enabled to support that, for the two groups consisting of subjects from 46 to 50 years, and of subjects from 71 to 79 years, a few links contribute in differentiating the behavior of males and females. The obtained results have been extensively commented on and discussed.

Regarding the model comparison analysis, the adopted strategy pointed out that, on average, the main discrepancies occur in four special brain connections, namely, the *left precuneus-right precuneus*, the *right middle temporal gyrus*, *posterior division–right central opercular cortex*, the *right middle temporal gyrus-right planum temporal posterior division* and the *Left Middle Temporal Gyrus*, *posterior division- Left Central Opercular Cortex*. Their possible role at resting state has been discussed. The paper is intended as a contribution in shedding more lights on the Neuroscience of resting state. Future perspective leads towards a deeper study of the principal networks such as DMN (Default Mode Network), SN (Salience Network), CEN (Central Executive Network) as well as the functional connectivity changing over the lifespan in patients affected by neuropsychiatric diseases.

## Methods

### Participants and MRI Data acquisition

The studied cohort consisted of 133 right-handed subjects, 51 males and 82 females. We clustered the sample in 14 age classes, ranging from 6 to 79 years of age, namely males and females were separately studied and divided into 14 separate 5-year-wide age groups (G1_M,F_ to G14_M,F_). Table 1 provides characteristics of the participants under investigation.

**Table 1:** 

Range of age (in y)	Id	# of males	# of females	# of subjects
6–10	1	7	7	14
11–15	2	3	2	5
16–20	3	1	2	3
21–25	4	6	16	22
26–30	5	5	7	12
31–35	6	4	4	8
36–40	7	3	7	10
41–45	8	4	2	6
46–50	9	3	6	9
51–57	10	3	7	10
58–65	11	3	5	8
66–70	12	2	5	7
71–75	13	5	10	15
76–79	14	2	2	4
Total		51	82	133

Groups formed by the subjects involved in the study.

None of the participants were taking psychoactive medications at the time of the scan or had a history of neurological or psychiatric disorders. According to the recommendations of the declaration of Helsinki for investigations on human subjects, the present study was specifically approved by the Ethics Committee of Don Gnocchi Foundation (Milan, Italy). Written informed consent from all subjects to participate in the study were obtained before study initiation.

### Data acquisition

Brain MR images were acquired using a 1.5 T scanner (Siemens Magnetom Avanto, Erlangen, Germany) with eight-channel head coil. rfMRI, BOLD EPI images (TR/TE = 2500/30 ms; resolution = 3.1 × 3.1 × 2.5 mm^3^; matrix size = 64 × 64; number of axial slices = 39; number of volumes = 160; flip angle = 70°; acquisition time = 6 min and 40 s) were collected at rest. Subjects were instructed to keep their eyes closed, not to think about anything in particular, and not to fall asleep. High-resolution T1-weighted 3D images (TR = 1900 ms; TE = 3.37 ms; matrix 192 × 256; resolution 1 × 1 × 1 mm^3^; 176 axial slices) were also acquired and used as anatomical references for rfMRI analysis.

### Image pre-processing

Pre-processing of rfMRI data was carried out using FSL [26], [27]. Standard pre-processing steps involved: motion correction, non-brain tissue removal, spatial smoothing with a 5 mm full width at half maximum Gaussian kernel, and high-pass temporal filtering with a cut-off frequency of 0.01 Hz. Subsequently, single-subject spatial ICA with automatic dimensionality estimation was performed using MELODIC [28] and FMRIB’s ICA-based Xnoiseifier (FIX, http://fsl.fmrib.ox.ac.uk/fsl/fslwiki/FIX) [29] was used to regress the full space of motion artifacts and noise components out of the data [30]. The training set for FIX was obtained using a separate group of HC (N = 42; age 35.7 ± 22.3 years; M/F = 19/23), whose data had been acquired using the same MRI protocol. After the pre-processing, each single-subject 4D dataset was first aligned to the subject’s high-resolution T1-weighted image using linear registration.

Participants were classified according to their age in 5 classes (5–10, 11–18, 19–40, 41–60, 61–79), and an age class-specific template representing the average T1-weighted anatomical image across subjects was built using the Advanced Normalization Tools (ANTs) toolbox [31]. Each participant’s cleaned dataset was co-registered to its corresponding structural scan, then normalized to the class-specific template before warping to standard MNI152 space, with 2×2×2 mm^3^ resampling. This step was undertaken to avoid the bias of trying to match brain of very different sizes to a single template.

### Computation of anatomical and functional matrices

For each subject, rfMRI data were parcelled into 94 cortical areas using the Harvard-Oxford Atlas (HOA) (see Table 2) [32], [33], [34], [35], then time-series were extracted from each one of these regions of interest. Subject-specific FC matrices were estimated by correlating each pair’s time-series. The resulting 94x94 matrix was used as functional connectivity matrix **F** whose entries are *F*_*ij*_.

**Table 2:** Nodes and correspondent cerebral areas.

Node	Cerebral area	Node	Cerebral area
1	Left Frontal Pole	48	Right Frontal Pole
2	Left Insular Cortex	49	Right Insular Cortex
3	Left Superior Frontal Gyrus	50	Right Superior Frontal Gyrus
4	Left Middle Frontal Gyrus	51	Right Middle Frontal Gyrus
5	Left Inferior Frontal Gyrus, pars triangularis	52	Right Inferior Frontal Gyrus, pars triangularis
6	Left Inferior Frontal Gyrus, pars opercularis	53	Right Inferior Frontal Gyrus, pars opercularis
7	Left Precentral Gyrus	54	Right Precentral Gyrus
8	Left Temporal Pole	55	Right Temporal Pole
9	Left Superior Temporal Gyrus, anterior division	56	Right Superior Temporal Gyrus, anterior division
10	Left Superior Temporal Gyrus, posterior division	57	Right Superior Temporal Gyrus, posterior division
11	Left Middle Temporal Gyrus, anterior division	58	Right Middle Temporal Gyrus, anterior division
12	Left Middle Temporal Gyrus, posterior division	59	Right Middle Temporal Gyrus, posterior division
13	Left Middle Temporal Gyrus, temporooccipital part	60	Right Middle Temporal Gyrus, temporooccipital part
14	Left Inferior Temporal Gyrus, anterior division	61	Right Inferior Temporal Gyrus, anterior division
15	Left Inferior Temporal Gyrus, posterior division	62	Right Inferior Temporal Gyrus, posterior division
16	Left Inferior Temporal Gyrus, temporooccipital part	63	Right Inferior Temporal Gyrus, temporooccipital part
17	Left Postcentral Gyrus	64	Right Postcentral Gyrus
18	Left Superior Parietal Lobule	65	Right Superior Parietal Lobule
19	Left Supramarginal Gyrus, anterior division	66	Right Supramarginal Gyrus, anterior division
20	Left Supramarginal Gyrus, posterior division	67	Right Supramarginal Gyrus, posterior division
21	Left Angular Gyrus	68	Right Angular Gyrus
22	Left Lateral Occipital Cortex, superior division	69	Right Lateral Occipital Cortex, superior division
23	Left Lateral Occipital Cortex, inferior division	70	Right Lateral Occipital Cortex, inferior division
24	Left Intracalcarine Cortex	71	Right Intracalcarine Cortex
25	Left Frontal Medial Cortex	72	Right Frontal Medial Cortex
26	Left Juxtapositional Lobule Cortex	73	Right Juxtapositional Lobule Cortex
	(formerly Supplementary Motor Cortex)		(formerly Supplementary Motor Cortex)
27	Left Subcallosal Cortex	74	Right Subcallosal Cortex
28	Left Para cingulate Gyrus	75	Right Para cingulate Gyrus
29	Left Cingulate Gyrus, anterior division	76	Right Cingulate Gyrus, anterior division
30	Left Cingulate Gyrus, posterior division	77	Right Cingulate Gyrus, posterior division
31	Left Precuneus Cortex	78	Right Precuneus Cortex
32	Left Cuneal Cortex	79	Right Cuneal Cortex
33	Left Frontal Orbital Cortex	80	Right Frontal Orbital Cortex
34	Left Parahippocampal Gyrus, anterior division	81	Right Parahippocampal Gyrus, anterior division
35	Left Parahippocampal Gyrus, posterior division	82	Right Parahippocampal Gyrus, posterior division
36	Left Lingual Gyrus	83	Right Lingual Gyrus
37	Left Temporal Fusiform Cortex, anterior division	84	Right Temporal Fusiform Cortex, anterior division
38	Left Temporal Fusiform Cortex, posterior division	85	Right Temporal Fusiform Cortex, posterior division
39	Left Temporal Occipital Fusiform Cortex	86	Right Temporal Occipital Fusiform Cortex
40	Left Occipital Fusiform Gyrus	87	Right Occipital Fusiform Gyrus
41	Left Frontal Operculum Cortex	88	Right Frontal Operculum Cortex
42	Left Central Opercular Cortex	89	Right Central Opercular Cortex
43	Left Parietal Operculum Cortex	90	Right Parietal Operculum Cortex
44	Left Planum Polare	91	Right Planum Polare
45	Left Heschl’s Gyrus (includes H1 and H2)	92	Right Heschl’s Gyrus (includes H1 and H2)
46	Left Planum Temporale	93	Right Planum Temporale
47	Left Occipital Pole	94	Right Occipital Pole

Brain HOA.

The HOA was also used to estimate the pairwise Euclidean distance between all the centroids of the regions of interest. The resulting values were used to define the 94x94 distance matrix **D**, whose elements *D*_*ij*_ appear in the exponent of the model (see Eq (3)).

## Model description

Following [20], we compute the weight of the edge between two nodes i and j as follows:

$$W_{ij}(t)=\beta (i,j,t_{fixed})\;e^{-(\eta {(t)D}_{ij}-\alpha (i,j,t))}.$$ (1)

Here, *t*_*fixed*_ denotes an instant corresponding to the interval during which data are recorded, while *t* is a time parameter spanning the whole life of the investigated neural network.

The function *β (i*, *j*, *t*_*fixed*_*)* depends on the topological growth of the neural network, and it can be approximated by the product of the degrees *deg(i)*, *deg(j)* of nodes *i* and *j*, respectively (see [20], [36]). We remind that the degree of a node is the number of edges connected to the node and that it is computed from the thresholded correlation matrix.

The parameter *η* is time-dependent and represents a kind of weight on the anatomical distances. In principle it changes with age (i.e. it is a function of t) [37], but, for simplicity, in this paper we have considered it constant, and consequently, it has been incorporated in the matrix **D** = [*Dij*]. Therefore, *η* reduces to a normalization factor introduced on the anatomical distances, namely, the entries of the distance matrix **D** range in the real interval [0,1].

The function *α(i*, *j*, *t)* relates to the functional connectivity, and it can be realistically modeled as the product of two functions *f (i*, *j)* and *g(t)*:

α(i,j,t)=f(i,j)g(t) (2)

where *f(i*, *j)* is the correlation between nodes *i* and *j* (depending on the task and/or resting state correlation), while *g(t)* is connected to stage of life in which the volunteer falls when the data are acquired. The function *α(i*, *j*, *t)* is directly available from the collected data, and, when there is no need of the synthetic decomposition provided by Eq (2), we simply denote by **F(t)** = [Fij(t)] the corresponding FC matrix (i.e., the correlation matrix) collected at time *t*.

As a consequence, the employed model can be rewritten and simplified as follows

$$W_{ij}(t)=\frac{\deg\left(i\right)\deg\left(j\right)}{W_{maz}(t)}e^{-(\eta D_{ij}-F_{ij}^{S}(t))},$$ (3)

where *W*_*max*_*(t)* is the normalization value, with *i* and *j* ranging from 1 to N, being N the total number of the areas of the chosen neural HOA, that is 94 in the present paper (see Table 2).

## Analytic description

First of all, let us comment briefly on the entries of the functional connectivity matrices made available by the data acquisition system. As usual, these consist of positive, zero and negative values. The problem of negative correlations is a very important issue, highly debated in Neuroscience. One of the advantages of the proposed model is that a positive weight can be assigned even to links characterized by negative correlations, so that these could be considered as well. However, since the main focus of the present paper is different, we have preferred to avoid including negative correlations in our discussions. Consequently, these entries have been set to zero, and our study is based on the resulting non-negative matrices.

We split the analysis of the available sample in two different steps, a background analysis, and a graph analysis.

1. The background analysis exploited, first of all, a recently proposed [23] double-step thresholding procedure. The first step is applied to the functional data made available by the acquisition system, and provides the functional correlations $F_{ij}^{S}(t)$ to be introduced in the exponent of the formula for the computation of the matrices *W*_*ij*_(*j*) according to the FD model (see (3)). The second step acts on the computed matrices *W*_*ij*_(*j*), and leads to the construction of a graph that can be realistically assumed as representative of the neural network having the strongest activity during the life span and at resting state.
2. The graph analysis is based on the previously determined representative graph, and introduces a new metric on the vertices, in order to favor links having endpoints of higher degrees. Indeed, as we have explained in the Introduction section, the leading idea behind the FD model is that the areas playing a central role during the brain activity can be better outlined, in our opinion, by focusing on nodes having high degrees. This results in refusing a few links previously selected (some of them even of high frequency all over the various groups) which consequently should be interpreted as an auxiliary indication of the brain activity at resting state.

### Background analysis

Let’s now explain in detail the two steps of the thresholding procedure applied to the considered sample.

#### First thresholding step

For each one of the 133 subjects, the distribution of the non-negative entries of the functional connectivity matrix **F**_**k**_ = [*F*_*k*_ (*i*, *j*, *t*)] (*k* ∊ {1, …,133}) has been organized in a histogram consisting of ten bins. For h∊ {1,…,10}, the h-th bin of each histogram covers the interval of intensities $\left[\frac{h-1}{10},\frac{h}{10}\right]$, and its height represents the number of entries in this range for the corresponding distribution.

The analysis of each histogram pointed out that most of the data always accumulate in the first two bins. This is not surprising since, being at resting state, the involved functional correlations are expected to be not so great. Since our aim was to focus on the links having strongest intensities, we defined data-depending thresholds that select, for each one of the 133 distributions, a suitable neighbor of interest (NOI) [23]. This is done by fixing a q-quantiles partition of each distribution, and by considering, for each *k* ∊ {1, …,133}, a reference quantile q_k_ such that all data above this quantile are contained above the second bin of the corresponding histogram. We recall that this implies that the (100(q-q_k_)/q)% of the data of the k-th distribution contributes to the corresponding NOI.

The resulting thresholded matrix **F**_**k**_**s** = [*F*_*k*_*s*(*i*, *j*, *t*)], *k* ∊ {1, …,133} is exploited for the computation of the node degrees of the k-th distribution, and then included in the exponent of the model Eq (3), together with the *D*_*ij*_ values computed as described previously. This provides the 133 matrices **W**_**k**_ = [*W(i*, *j*, *t*, *k)*].

Then, according to their age, the 133 subjects have been split into 28 5-years ranged different groups, 14 formed by males and denoted by *M*_*i*_, and 14 formed by females and denoted by *B*_*i*_, *i* ∊ {1, …,14} (see Table 1). Within each group *M*_*i*_ (*i*∈ {1, …, 14}), the first-step thresholded matrices associated to the corresponding subjects are summed up, and then divided by the cardinality of the group, so encoding in a single matrix $\bar{W_{M_{i}}(t)}$ the information concerning the whole group. The same procedure has been applied to *B*_*i*_, which associates a single matrix $\bar{W_{B_{i}}(t)}$ to each one of the 14 groups of females.

#### Second thresholding step

The second thresholding step aims in preserving, among the previously selected ones, the links having higher intensities [23].

To this, we apply, for each *i*, a second threshold *λ*_*i*_ on $\bar{W_{M_{i}}(t)}$ (respectively *μ*_*i*_ for $\bar{W_{B_{i}}(t)}$). In order to compute *λ*_*i*_ (respectively *μ*_*i*_), we have organized all the entries of the first-step thresholded matrices in a new histogram, consisting of 10 bins, and then we have considered the center *m*_*i*_ (respectively *b*_*i*_) of the bin formed by the highest 10% of entries. Then, the thresholds *λ*_*i*_ (respectively *μ*_*i*_) should be assumed to be equal to some suitable percentage of *m*_*i*_ (respectively *b*_*i*_). Since no specific knowledge in advance on the sample was available, it seemed to us quite reasonable to consider *λ*_*i*_ = 1/2 *m*_*i*_ (respectively *μ*_*i*_ = 1/2 *b*_*i*_), setting to 0 each entry below the thresholds.

The above choices are motivated by the fact that, working at resting state, we are interested in selecting only links having very high intensity. Actually, by using a greater percentage we would include very low intensities, which could be highly affected by noise. On the other hand, working with less than 10% of the highest entries could result in the loss of some link that, though not so strong, have a relevant importance at resting state.

Due to the resting state condition, we expect that the range of such highest intensities spans also some values that are not so strong. Indeed, this is the case, since, all over the 28 working groups, we found intensities even around 0.1, which, however, are not so common (see Table 3, Table 4 and Table 5).

**Table 3:** 

MALES		FEMALES	
Link	Frequency	Link	Frequency
**31–78**	14	**22–31**	14
**29–76**	13	**31–78**	14
**30–77**	13	**29–76**	13
**22–31**	12	**1–48**	11
**69–78**	12	**69–78**	12
**28–75**	11	**30–77**	10
**54–64**	11	**36–83**	9
**1–48**	10	**22–78**	9
**36–83**	8	**47–94**	9
**47–94**	8	**30–31**	8
**75–76**	7	**28–75**	8
**77–78**	7	**75–76**	7
**30–31**	7	**48–75**	7
**48–75**	7	**77–78**	7
**22–69**	6	**30–78**	6
**24–36**	5	**31–69**	6
**22–78**	5	**1–28**	5
**31–69**	4	**22–69**	5
**31–77**	4	**28–29**	3
**28–29**	4	**28–76**	2
**23–47**	3	**69–70**	1
**1–28**	3		
**29–75**	3		
**21–22**	2		
**28–48**	2		
**30–78**	2		
**22–77**	1		

The set *L* of emerging links and their presence over M_i_ and B_i_, i = 1,…,14. See also Table 2 for the matching with the corresponding cerebral areas.

**Table 4:** 

Link	M_1_	M_2_	M_3_	M_4_	M_5_	M_6_	M_7_	M_8_	M_9_	M_10_	M_11_	M_12_	M_13_	M_14_
**31–78**	0.41	0.58	0.73	0.47	0.50	0.6	0.79	0.73	0.62	1.00	0.68	0.67	0.90	0.90
**29–76**	0.40	0	0.64	0.43	0.30	0.52	0.68	0.19	0.50	0.80	0.26	0.43	0.52	0.61
**30–77**	0.29	0.30	0.44	0.34	0	0.37	0.47	0.39	0.40	0.74	0.29	0.40	0.41	0.76
**22–31**	0.26	0.35	0	0	0.34	0.24	0.40	0.32	0.28	0.35	0.42	0.44	0.53	0.59
**69–78**	0.29	0.32	0.41	0.28	0	0.32	0.53	0.40	0.20	0.69	0.43	0.41	0.57	0
**28–75**	0.53	0	0.50	0.37	0.23	0.34	45	0	0	0.61	0.19	0.29	0.43	0.38
**54–64**	0.28	0.25	0.58	0.30	0.29	0.47	0.52	0	0.40	0.45	0.40	0	0.34	0
**1–48**	0.42	0	0.43	0.44	0.22	0.31	0.43	0.08	0	0	0.21	0	0.43	0.50
**36–83**	0	0.23	0	0.33	0.44	0.46	0.59	0.34	0	0.50	0	0	0.38	0
**47–94**	0	0	0	0.34	0.30	0.47	0	0.43	0.32	0.36	0	0.38	0	0.52
**75–76**	0.26	0	0.46	0.29	0	0.35	0	0	0.18	0.56	0	0.31	0	0
**77–78**	0	0.24	0	0	0	0.37	0.43	0.19	0	0.34	0.23	0	0	0.62
**30–31**	0	0.25	0	0.27	0	0.30	0	0.17	0	0	0.20	0.30	0	0.46
**48–75**	0.34	0	0	0.34	0	0.28	0	0	0	0.50	0	0.29	0.39	0.39
**22–69**	0	0	0	0	0.21	0.27	0	0.13	0	0	0.21	0.41	0.40	0
**24–36**	0	0	0	0	0	0.29	0.39	0.15	0.30	0.43	0	0	0	0
**22–78**	0	0.17	0	0	0	0	0	0.24	0	0	0.22	0	0.39	0.34
**31–69**	0	0.23	0	0	0	0	0.44	0	0	0	0.36	0.37	0	0
**31–77**	0	0.25	0	0	0	0.30	0	0	0	0	0.22	0	0	0.49
**28–29**	0.29	0	0	0	0	0	0	0	0.19	0.50	0	0	0.40	0
**23–47**	0	0.16	0	0	0	0	0	0	0	0	0	0.29	0.38	0
**1–28**	0.27	0	0	0.28	0	0	0	0	0	0	0	0	0	0.36
**29–75**	0.28	0	0	0	0	0	0	0	0	0.47	0	0.27	0	0
**21–22**	0	0.22	0	0	0	0	0	0	0	0	0	0	0.36	0
**28–48**	0.27	0	0	0	0	0	0	0	0	0	0	0	0	0.35
**30–78**	0	0	0	0	0	0	0	0.12	0	0	0	0	0	0.51
**22–77**	0	0	0	0	0	0	0	0	0	0	0.19	0	0	0

Males. Functional connectivity intensities of the emerging links all over the 14 groups.

**Table 5:** 

Link	F_1_	F_2_	F_3_	F_4_	F_5_	F_6_	F_7_	F_8_	F_9_	F_10_	F_11_	F_12_	F_13_	F_14_
**22–31**	0.51	0.27	0.27	0.26	0.20	0.26	0.28	0.21	0.11	0.26	0.28	0.39	0.22	0.21
**31–78**	0.91	0.45	0.49	0.48	0.60	0.50	0.44	0.26	0.13	0.44	0.62	0.55	0.34	0.33
**29–76**	1	0.20	0.21	0.15	0.48	0.28	0.29	0	0.05	0.25	0.58	0.45	0.16	0.16
**1–48**	0.67	0	0	0.21	0.39	0.31	0.31	0.19	0.09	0.26	0.38	0.37	0.23	0.24
**69–78**	0	0.26	0.34	0.22	0.25	0.32	0.26	0	0.09	0	0.34	0.34	0.21	0.24
**30–77**	0.84	0.23	0	0.22	0.39	0	0.20	0.14	0	0.34	0.34	0.43	0.18	0
**36–83**	0.54	0	0.34	0.25	0.29	0	0.14	0	0.08	0.20	0.26	0.30	0	0
**22–78**	0	0.17	0.16	0.17	0.19	0.17	0.21	0	0.06	0.09	0	0	0.13	0
**47–94**	0	0	0.27	0.17	0.33	0.16	0.11	0.23	0	0.20	0.27	0	0.15	0
**30–31**	0	0.22	0.16	0.13	0.19	0	0.11	0.17	0	0	0	0.27	0.17	0
**28–75**	1	0	0	0.16	0.22	0.23	0.09	0	0	0.13	0.29	0.25	0	0
**75–76**	0.67	0	0	0	0.16	0.19	0.08	0	0	0.11	0.40	0.31	0	0
**48–75**	0.70	0	0	0	0.16	0.18	0	0	0	0.11	0.34	0.27	0.17	0
**77–78**	0	0.19	0	0	0.24	0	0.18	0	0	0.16	0.32	0.30	0.13	0
**30–78**	0	0.17	0.16	0	0.18	0	0.09	0	0	0.10	0	0	0.14	0
**31–69**	0	0.16	0.28	0.14	0	0.16	0	0	0	0	0	0.30	0	0.22
**1–28**	0	0	0	0	0.20	0.18	0	0	0	0.10	0.26	0	0	0.21
**22–69**	0	0.16	0	0.18	0	0.15	0	0	0.08	0	0	0	0	0.22
**28–29**	0.53	0	0	0	0	0	0	0	0	0	0.25	0	0	0.13
**28–76**	0	0	0	0	0	0	0	0	0	0	0.25	0	0	0.12
**69–70**	0	0	0	0	0	0	0	0	0	0	0	0	0	0.14

Famale. Functional connectivity intensities of the emerging links all over the 14 groups.

The resulting outputs consist of matrices ${\bar{W_{M_{i}}(t)}}^{*}$ and ${\bar{W_{B_{i}}(t)}}^{*}$, *i* ∊ {1, …, 14}, where the * denotes the second thresholding step.

#### Example

The group *M*_6_, namely the 6^*th*^ group of males, consists of 4 subjects (see Table 1). The matrix $\bar{W_{M_{6}}(t)}$ that represents the group is obtained by averaging the matrices *W*_*k*_ computed when *k* assumes the four values corresponding to the subjects in *M*_6_, namely $\bar{W_{M_{6}}(t)}=\frac{1}{4}\sum_{i=1}^{4}W_{i}^{s}(t)$.

**Fig 1:**
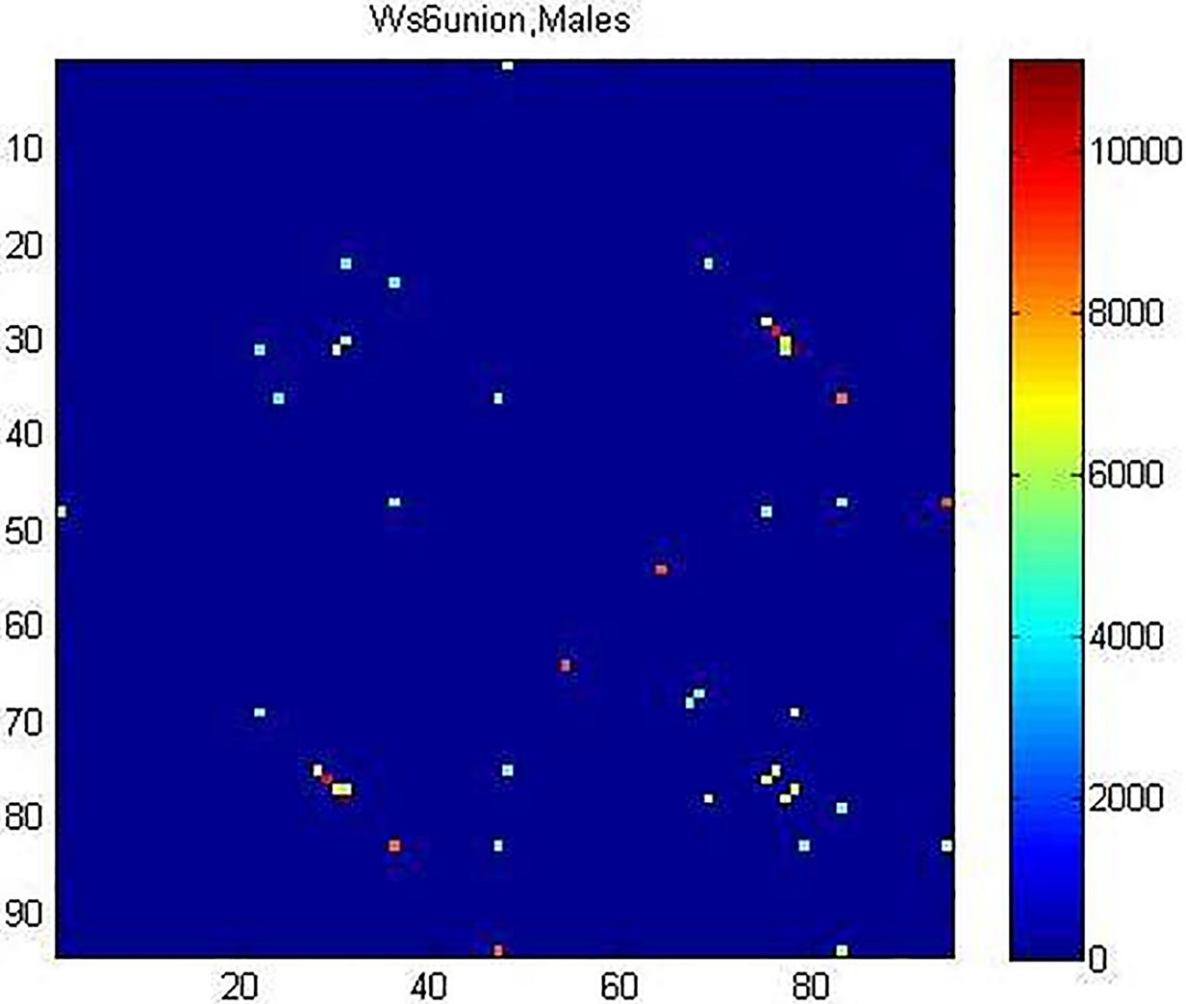
The thresholded matrix ${\bar{W_{M_{6}}(t)}}^{*}$ representing the group M_6_.

The center value *m*_6_ of the bin formed by highest 10% of its entries is determined. It results in *m*_*6*_ = 0.3848. Then the matrix $\bar{W_{M_{6}}(t)}$ is thresholded, by setting its entries equal to zero when these are below λ_6_ = 1/2 *m*_6_ = 0.1924. The resulting 94x94 sized matrix ${\bar{W_{M_{6}}(t)}}^{*}$ is represented in Fig 1, where different colors denote different functional intensities.

### Analysis to define the neural graph at rest

We denoted by *L* the set of *emerging links*, namely the set of all links related to non-zero entries in the 28 matrices (the 14 ${\bar{W_{M_{i}}(t)}}^{*}$ and the 14 ${\bar{W_{B_{i}}(t)}}^{*}$) provided by the FD model. This set *L* is collected in Table 3, where, for each link, the corresponding frequency, ranging from 1 to 14, is also reported. For instance, the frequency 11 associated to the link 28–75 in the male column means that this link has been selected by the FD model in 11 over the 14 male groups. Table 4 and Table 5 show the corresponding functional intensities all over the various groups.

**Fig 2:**
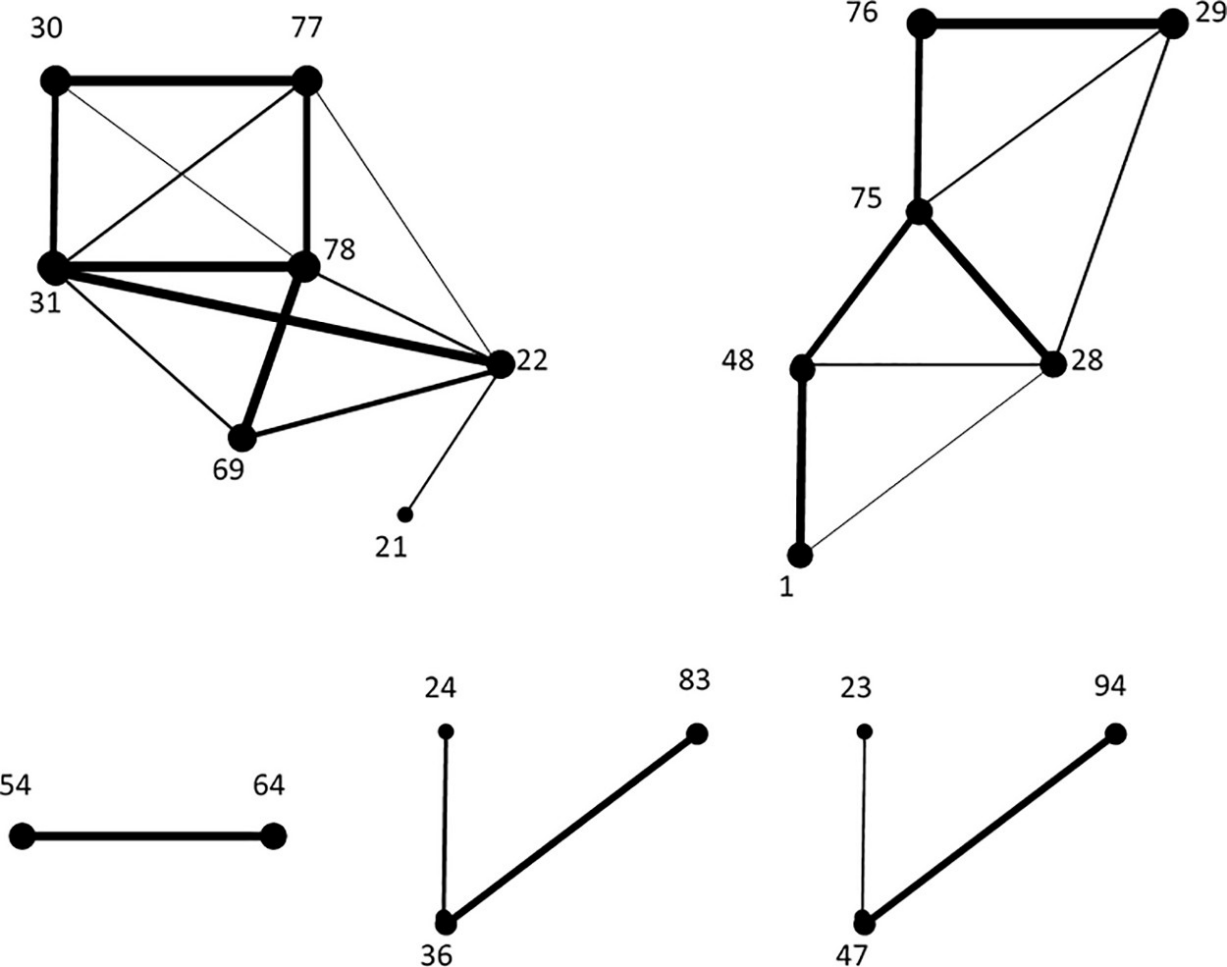
The representative graph of the neural network for males, as determined by the emerging links according to the FD model.

**Fig 3:**
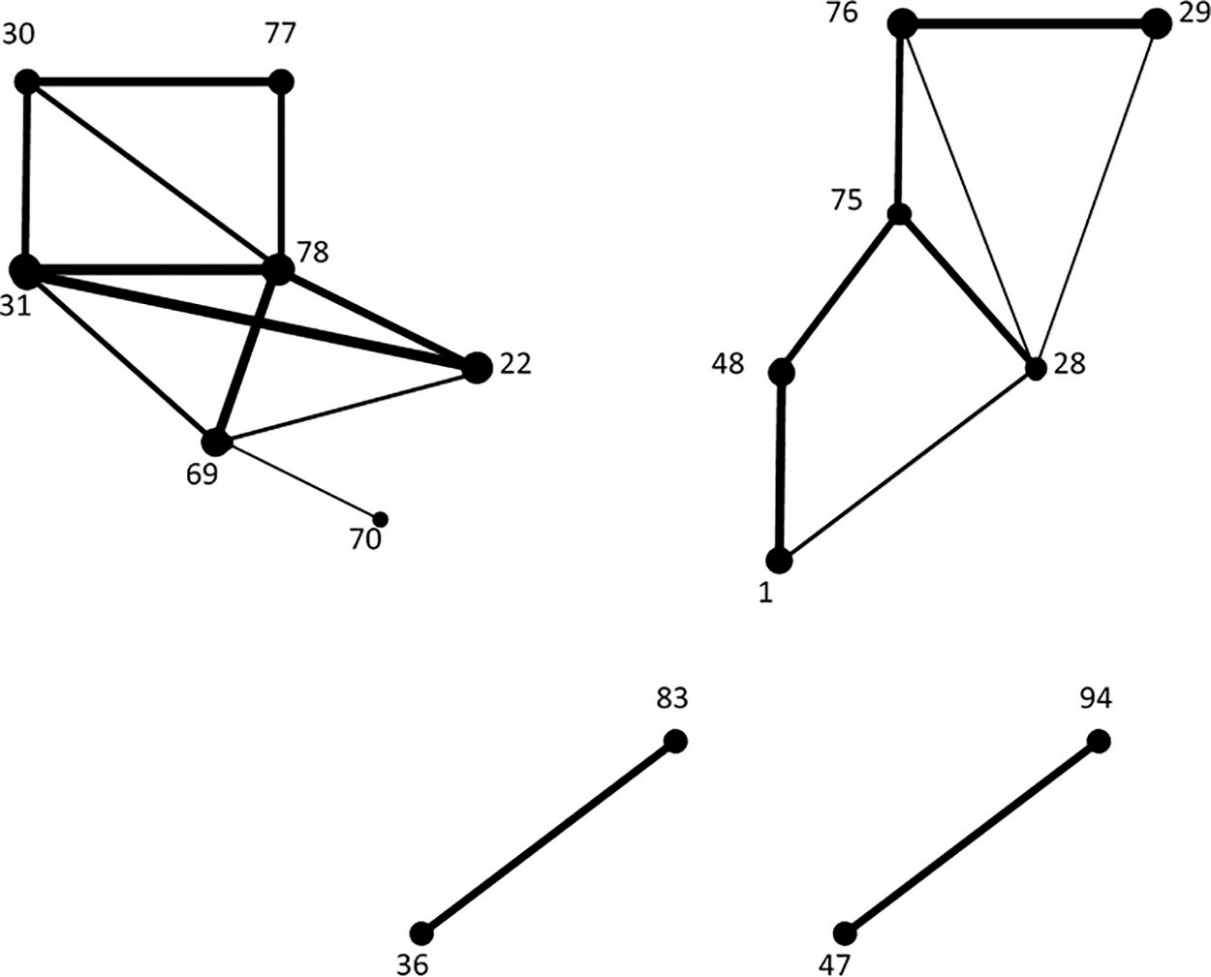
The representative graph of the neural network for males, as determined by the emerging links according to the FD model.

The male and female sets of emerging links provided by the FD model can be immediately turned in graphs, that can be realistically assumed as representative of the emerging neural network during the life span and at resting state. These are represented in Fig 2 and Fig 3, respectively, where the thickness of a link corresponds to its frequency of appearance over the life span.

One of the ideas behind the FD model is to favour the selection of links whose endpoints have higher degrees, since, in our opinion, this is preferable in view of understanding the areas playing a central role during the brain activity. In this view, a first glance to Fig 2 and Fig 3 would immediately lead us to remove some links, for example, 54–64 for males, or 36–83 and 47–94 for females. However, we preferred to implement an explicit methodology to catch such less meaningful links.

To this, we have deepened our approach to the analysis of the representative graph by associating to each node *v* of the HOA (see Table 2) a new topological metric, the *Centrality Index* of *v*, hereinafter denoted by *CI(v)*. For the male (resp. female) sample, *CI(v)* is the normalized sum of the number of non-zero entries that involve v over the 14 matrices associated to the 14 male (resp. female) groups. That is, *CI(v)* is the sum of the degrees of *v* all over the corresponding 14 networks, divided by the greatest obtained values. Note that *CI(v)* is defined only for the nodes corresponding to non-zero lines of the double-step thresholded matrices.

Now, the selection from *L* of the links of interest is based on the following five steps.

### 1. Selection of the set of principal vertices

We have organized the distribution of the centrality indices in a histogram consisting of ten bins. The computation of the center of the 5^th^ bin provides 0.46, both for males and for females.

The set *V’* of principal vertices has been obtained by extracting from the HOA the nodes having centrality index greater than, or equal to, this value, namely:

$$V^{\prime }=\{v\in HOA,\;CI(v)\geq 0.46\}$$ (4)

Table 6 shows the distribution of the centrality indices for males and for females, where the vertices forming the set *V’* have been reported in the shaded cells.

**Table 6:** 

MALES				FEMALES			
Node	CI	Node	CI	Node	CI	Node	CI
**31**	1.00	73	0.12	**78**	1	71	0.08
**78**	0.98	79	0.12	**31**	0.88	32	0.06
**75**	0.73	26	0.10	**22**	0.63	43	0.06
**77**	0.66	32	0.10	**69**	0.53	46	0.06
**22**	0.63	70	0.10	**48**	0.51	12	0.04
**28**	0.56	50	0.07	**30**	0.49	25	0.04
**76**	0.56	65	0.07	**76**	0.49	42	0.04
**30**	0.54	71	0.07	**75**	0.47	67	0.04
**69**	0.54	3	0.05	1	0.39	68	0.04
**29**	0.49	21	0.05	28	0.39	72	0.04
**48**	0.49	45	0.05	77	0.37	80	0.04
36	0.41	51	0.05	29	0.35	86	0.04
47	0.39	57	0.05	47	0.33	18	0.02
64	0.39	67	0.05	83	0.31	20	0.02
83	0.39	68	0.05	94	0.27	21	0.02
1	0.37	8	0.02	36	0.24	26	0.02
54	0.37	10	0.02	64	0.22	27	0.02
40	0.27	12	0.02	87	0.22	39	0.02
94	0.24	39	0.02	54	0.2	45	0.02
87	0.22	42	0.02	70	0.2	55	0.02
7	0.20	46	0.02	17	0.18	57	0.02
24	0.20	55	0.02	23	0.16	59	0.02
17	0.17	59	0.02	7	0.14	63	0.02
23	0.15	93	0.02	40	0.14	66	0.02
				13	0.1	73	0.02
				79	0.1	74	0.02
				10	0.08	90	0.02
				24	0.08	93	0.02

Distribution of the centrality indices. The set V’ of the principal vertices is formed by the nodes in the shaded cells.

### 2. Selection of the principal links

The first set of edges to be included in the representative graph has been obtained by considering all links having non-zero occurrence in the matrices provided by the FD models, and whose endpoints both belong to the set *V’* previously introduced. That is, for any pair of vertices *a*,*b* ∈ *V*′ we selected the link *a* − *b* if and only if it appeared in at least one of the matrices associated to the considered groups (14 for males and 14 for females). We denoted by *E’* this set of edges.

### 3. Secondary vertices

At this point we added to *V’* further nodes, having a “strong” link with some node of *V’*. More precisely, a node $\bar{v}\notin V’$ is considered, if and only if there exists a node *v* ∈ *V*′ such that the link $\bar{v}-v$ appears in at least 7 of the 14 matrices representing the groups (*Mi* and *Fi*, respectively, *i* = 1, …, 14). This forms the set

$$V^{\prime \prime }=\left\{\bar{v},\;\exists \;v\in V^{\prime },\left|\bar{v}-v\right|\geq 7,\bar{v}\notin V^{\prime }\right\},$$ (5)

where the symbol | | stands for the cardinality.

### 4. Secondary edges

The set *V”* naturally leads to consider also the set of edges *E”* consisting of all links $\bar{v}-v$ provided by Eq (5), which deserve to be considered due to their high frequency. However, it is worth to be included also possible links provided by the FD model, having non-zero frequency and both endpoints $\bar{v},v\in V^{\prime \prime }$. Note that, though their frequency is not necessarily at least 7 (see Table 3), such links deserve to be considered as well, since further connections between pairs of nodes in *V”* relate immediately to an increasing robustness of the neural network.

#### The representative graph

As a result of the above five items, we have assumed the neural network at resting state represented by the graph *G(V*,*E)* such that *V* = *V*′ ∪ *V*" and *E* = *E*′ ∪ *E*".

Table 7 shows the sets *V’ a*nd *V”* for the male and female groups.

**Table 7:** 

**Males**	V’ = {22, 28, 29, 30, 31, 48, 69, 75, 76, 77, 78}	V” = {1}
**Females**	V’ = {22, 30, 31, 48, 69, 75, 76, 78}	V” = {1, 28, 29, 77}

The sets of nodes V^***’***^ and V^***”***^ for males and females.

Notably, while *V’* and *V”* have a different structure in males and females, their union *V*′ ∪ *V*" becomes the same for both samples. Table 8 provides the list of all selected edges *E*, listed according to their frequency of appearance. In red and in blue are represented, respectively, the links forming the subsets *E’* and *E”* involved in *G*(*V*, *E*), while in black the removed emerging links. See also Table 2 for the matching with the corresponding cerebral areas.

**Table 8:** 

MALES		FEMALES	
Link	Frequency	Link	Frequency
**31–78**	14	**22–31**	14
**29–76**	13	**31–78**	14
**30–77**	13	**29–76**	13
**22–31**	12	**1–48**	11
**69–78**	12	**69–78**	12
**28–75**	11	**30–77**	10
**54–64**	11	**36–83**	9
**1–48**	10	**22–78**	9
**36–83**	8	**47–94**	9
**47–94**	8	**30–31**	8
**75–76**	7	**28–75**	8
**77–78**	7	**75–76**	7
**30–31**	7	**48–75**	7
**48–75**	7	**77–78**	7
**22–69**	6	**30–78**	6
**24–36**	5	**31–69**	6
**22–78**	5	**1–28**	5
**31–69**	4	**22–69**	5
**31–77**	4	**28–29**	3
**28–29**	4	**28–76**	2
**23–47**	3	**69–70**	1
**1–28**	3		
**29–75**	3		
**21–22**	2		
**28–48**	2		
**30–78**	2		
**22–77**	1		

Emerging links *L* and their presence over M_i_ and B_i_, i = 1,…,14. The links belonging to E^’^ are shown in red while the links belonging to E‘‘ in blue. The black links do not contribute to the relevant network.

**Fig 4:**
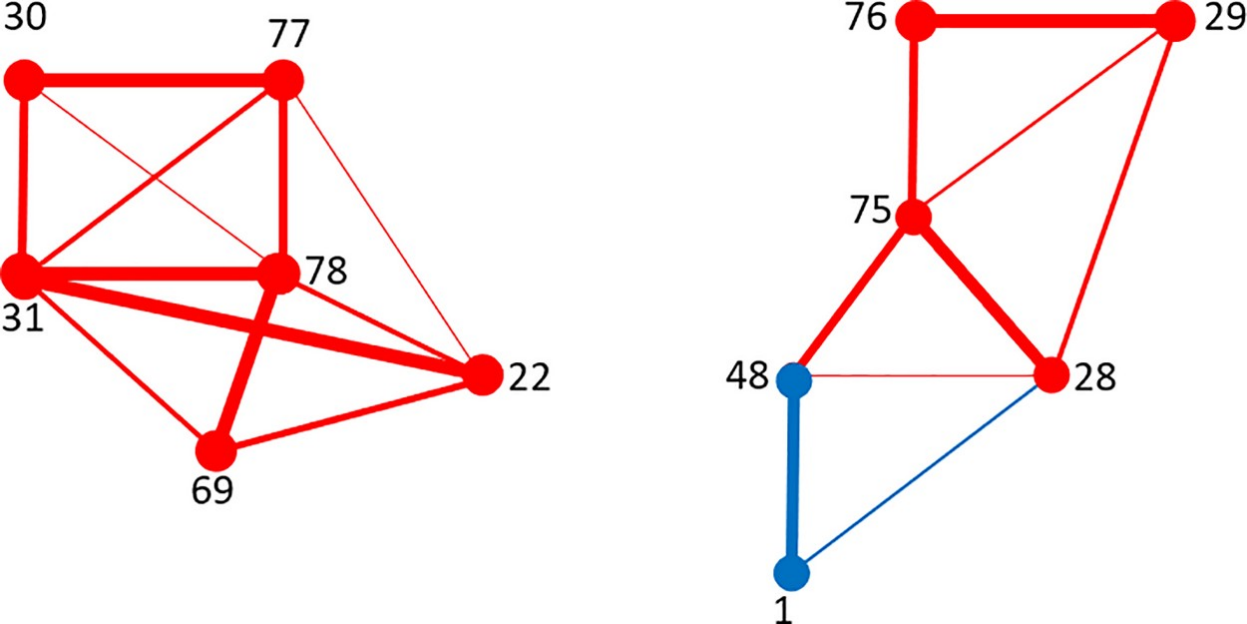
Males. The representative graph *G(V*,*E)*.

**Fig 5:**
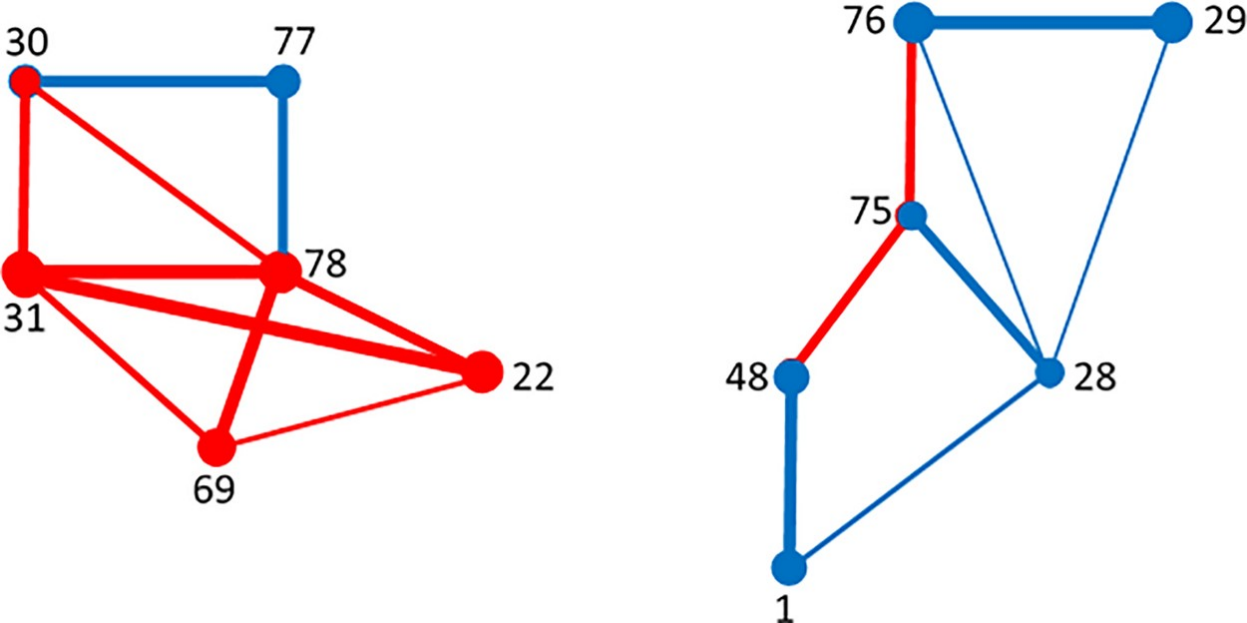
Females. The representative graph *G(V*,*E)*.

Fig 4 and Fig 5 show the graph *G*(*V*, *E*) for males and for females, respectively. Also comparing the known literature concerning the resting state (see for example [25], [38], [39]), we are leading to interpret *G*(*V*, *E*) as the Default Mode Network (DMN).

### Discussion on the representative graph

We have exploited the recently proposed FD model [20] for the construction of a representative graph *G(V*,*E)* of the neural network, over the life span and at resting state. The set *E* of edges, and the corresponding set *V* of vertices, has been selected from the thresholded matrices provided by the FD model.

We can see that the representative graph largely overlaps with the nodes and links within the DMN. In this sense we referred to Raichle et al. [6], [7], Greicius et al. [25] and Buckner et al. [12], that defined the DMN as composed by specific areas such as the prefrontal gyrus, the ventral precuneus, the posterior cingulate gyrus and the bilateral inferior parietal regions, the angular gyrus, the temporoparietal junction, the lateral temporal cortex and part of the temporal lobe. We found that, both for males and for females, and over the range of age considered in this study, a main role seems to be played by the precuneus, the cingulate gyrus, the frontal pole, the paracingulate gyrus, and the occipital cortex, which are characterized by high functional strength and degree. In particular, if we focus our attention on the precuneus, cingulate gyrus and frontal pole such characteristics are consistent with their role as hubs within the DMN. This is not surprising since the DMN is most active when the brain is at rest, while it deactivates when the brain is directed outwards or engaged in a cognitive task [12], [13].

Although we consider the Euclidean distance between cerebral areas, our findings fit with the results in [40], where a general correspondence between functional and structural connectivity has been demonstrated across the cortex, highlighting that the structural core contains many connecting hubs, and a central role seems to be played by the right and left posterior cingulate cortex, in other sub-divisions of the cingulate cortex, in the precuneus and in the cuneus.

We would like to stress that we considered an anatomical atlas (Harvard-Oxford) when we started our analysis. This was part of the set experimental paradigm. Nevertheless, the FD model can be applied to different structural data (e.g. DTI and tractography) referring to particular atlases (e.g. the Destrieux Atlas [24]) as well as to every functional data (fMRI, EEG, MEG,…). To this, the formula in Eq (3) should be considered independently of the methods employed to collect the corresponding data, even if, of course, the resulting numerical values must be interpreted accordingly.

Even more detailed atlases, such as the ones that combine functional (for instance rsfMRI) and structural (DTI) datasets, would surely increase the precision of the outcomes.

### Mathematical discussion

Looking at the provided outputs, we can see that both the graphs representing the neural network at resting state for males and females consist of two connected components, and each of them is a planar graph. A graph *G* = *(V*, *E)* is planar if it can be “drawn” on the plane without edges crossing except at endpoints. In other words, a planar graph is a graph that can be embedded in the plane. More precisely, there is a 1 to 1 function *f*: *V* → *R*^2^ and for each *e* ∈ *E* there exists a 1 to 1 continuous function *ge*, *ge*: [0, 1] → *R*^*2*^ such that:

1. *e* = *xy* implies *f*(*x*) = *g*_*e*_(0) and *f*(*y*) = *g*_*e*_(0).
2. *e* ≠ *e*′ implies that *g*_*e*_(*x*) ≠ *g*_*e*′_(*x*′) for all *x*,*x*′ ∈ (0,1).

For a given set of nodes, an increasing number of links tends to reduce the probability that the graph is planar. Also, increasing the number of links reflects in increasing the cost of the graph. On the other side, the presence of more paths between a same pair of nodes improves the resistance of the networks to random attacks, so preserving the efficiency in transmitting information. Therefore, the planar structure of our outputs, and the presence of cycles in both of them, suggests a tendency of the brain to reduce, but not minimize, the cost, and a kind of robustness of the DMN.

## Statistical analysis of results

### Objectives of the analysis

In the previous section we have provided a graph representation of the human connectivity, as it emerges at resting state, by exploiting the FD model and the related thresholding procedure. As we have previously detailed, the set *E* of edges of the resulting graph *G(V*,*E)* has been selected from the set *L* of all the emerging links as detailed previously

The aim of the statistical analysis reported in this section is to investigate the differences in connectivity between males and females in the different age classes. In detail, we want to investigate whether the set *L* of emerging links is able to discriminate between males and females. If such discrimination is possible, we aim at selecting the specific age groups that present differences between males and females (**global analysis**), and for such age groups, the specific links that better discriminate males and females (**local analysis**). The two objectives of this analysis are pursued in two separate phases and are addressed by means of different statistical inferential methods.

#### Global analysis

In the first phase, we test if, globally, the functional connectivity of males is different with respect to the one of the females. The performed test is a global test, in the sense that it considers the functional connectivity of all emerging links together. In a preliminary study that is not reported here for brevity, we tested if mean and variance of the signal *W* depend on age. We found out that—while there is no significant effect of age on the mean connectivity—there is a significant effect of age on the variance. Hence, we decided to run a separate analysis for each age group.

The method used in this phase is a non-parametric multivariate analysis of variance (MANOVA).

#### Local analysis

If on a specific age group we observe significant differences between males and females (i.e., if the global MANOVA test is significant for that class), then it is of great interest to select the specific links which significantly discriminate males from females. Hence, in the second phase of the analysis within the age groups selected in the first phase, we test if locally the functional connectivity of males is different with respect to the one of females. The performed test is a local test since it is performed for every link separately and provides a selection of the statistically significant links. The method used in this phase is a non-parametric univariate analysis of variance (ANOVA).

### Statistical methods

Let *L*_*M*_ and *L*_*B*_ denote the sets of the links emerging from the previous analysis for males and females, respectively (i.e., the links reported in Table 3, left and right columns, respectively). Since we are interested in comparing the connectivity of males and females, it is natural to compare it on a common set of links. Hence, the analysis that we carry on is on the comparison between the connectivity of males and females on the set *L = L*_*M*_ *U L*_*B*_ (where *U* denotes the operation of union of the two sets of links). Let us denote by *Q =* |*L*| the cardinality of the set *L*. Note that in our case we have a total of *Q =* 29 links emerging from the previous analysis. The aim of the statistical analysis performed here is then to test for differences between the connectivity of males and females across the set of emerging links within each group of age (see Table 8).

First of all, note that the number of individuals in the different groups of age is in some cases very small, with some groups containing just one or two individuals. For such reasons, to increase the sample size before performing the statistical analysis, we decided to merge the original 14 age groups *M*_*i*_ and *B*_*i*_ into the seven groups *M*_*i’*_ and *B*_*i’*_, reported in Table 9 (with the new index *i’* ranging from 1 to 7). The following global and local analyses are carried out on the new groups.

**Table 9:** 

Original Groups	New Groups	i'	# of males	# of females
6–10	6–15	1	10	9
11–15				
16–20	16–21	2	7	18
21–25				
26–30	26–35	3	9	11
31–35				
36–40	36–40	4	7	9
41–45				
46–50	46–50	5	6	13
51–57				
58–65	58–70	6	5	10
66–70				
71–75	71–79	7	7	12
76–79				

Ages and sample sizes regarding the subjects involved in the study of the original groups, and of the new groups after merging into seven classes.

### Global analysis

The aim of the global analysis is to investigate the differences between males and females in terms of functional connectivity, for every separate age group. Formally, *∀i*′ = 1,…,7, let $W_{M_{{i\prime }_{k}}}$ (with *k* = 1,…,|*M*_*i*′_|) denote the functional connectivity matrices of the |*M*_*i*′_| male subjects of the group *M*_*i*′_. Analogously, *∀i*′ = 1,…,7, let $W_{B_{{i\prime }_{k}}}$ (with *k* = 1,…,|*B*_*i*′_|) denote the functional connectivity matrices of the |*B*_*i*′_| female subjects of the group *B*_*i*′_. We are interested in testing the differences between the two *Q*-variate samples $\{{[W}_{M_{{i^{\prime }}_{1}}}{]}_{e,}{[W}_{M_{{i^{\prime }}_{2}}}{]}_{e}\;,\ldots ,[W_{M_{{i^{\prime }}_{{|M}_{i^{\prime }}|}}}{]}_{e}{\}}_{e\in E}$ and $\{{[W}_{B_{{i^{\prime }}_{1}}}{]}_{e,}{[W}_{B_{{i^{\prime }}_{2}}}{]}_{e}\;,\ldots ,[W_{B_{{i^{\prime }}_{{|B}_{i^{\prime }}|}}}{]}_{e}{\}}_{e\in E}$, for all links *e* belonging to *L*. For easier notation, let us denote with $\left(y_{{i^{\prime }}_{1}},y_{{i^{\prime }}_{2}},\ldots ,y_{{i^{\prime }}_{{|M}_{i^{\prime }}|}}\right)$ and $\left(x_{{i^{\prime }}_{1}},x_{{i^{\prime }}_{2}},\ldots ,x_{{i^{\prime }}_{{|B}_{i^{\prime }}|}}\right)$ the two samples of males and females, respectively, of group *i’*. Note that, for all *k*, $y_{{i^{\prime }}_{k}}$ and $x_{{i^{\prime }}_{k}}$ are random vectors of dimension *Q* containing the entries of the *Q* emerging links. Assume that *∀i*′ = 1,…,7: $y_{{i^{\prime }}_{k}}∼i.i.d.Y_{i^{\prime }}$, and $x_{{i^{\prime }}_{k}}∼i.i.d.X_{i^{\prime }}$, where ***Y***_*i*′_ and ***X***_*i*′_ are two independent *Q-*variate random vectors and *i*.*i*.*d*. is the acronym for *independent and identically distributed*.

For all *i’ = 1*,*…*,*7* we aim at testing the null hypothesis of equality between the distributions of ***Y***_*i*′_ and ***X***_*i*′_ against the alternative hypothesis of a different distribution between the two variables:

$$H_{0_{i^{\prime }}}:Y_{i^{\prime }}=X_{i^{\prime }}\;\mathrm{against}\;H_{1_{i^{\prime }}}:Y_{i^{\prime }}\neq X_{i^{\prime }}.$$ (6)

The test (6) is a multivariate test since it involves the distribution of the *Q*-dimensional random vectors ***Y***_*i*′_ and ***X***_*i*′_. In case of rejection of the null hypothesis for group *i’*, we will say that we have enough evidence to state that there is a significant difference between males and females for that particular age group. In addition, note that for all age groups, the dimension of the random vectors *Q* = 29 is higher than the sample sizes (last two columns of Table 9). Hence, it is not possible to employ classical parametric statistical methods to perform the test (6), since they typically require that |*M*_*i*′_|+|*B*_*i*′_| > *Q*. For such reason, we perform a non-parametric permutation test based on permutations of the observations over the two groups and on a direct combination of the classical ANOVA test statistic over all *Q* links in *E* [41]. Specifically, if we now fix a link *e* ∈ *L*, we can compute the ANOVA test statistic for testing differences between males and females on the link *e* [42]:

$${\left(T_{i^{\prime }}\right)}_{e}=\frac{\left|M_{i^{\prime }}\right|{\left[{\left({\bar{y}}_{i^{\prime }}\right)}_{e}-{\left({\bar{\bar{m}}}_{i^{\prime }}\right)}_{e}\right]}^{2}+\left|B_{i^{\prime }}\right|{\left[{\left({\bar{x}}_{i^{\prime }}\right)}_{e}-{\left({\bar{\bar{m}}}_{i^{\prime }}\right)}_{e}\right]}^{2}}{\left[\sum_{k=1}^{\left|M_{i^{\prime }}\right|}{\left[{\left(y_{{i^{\prime }}_{k}}\right)}_{e}-{\left({\bar{y}}_{i^{\prime }}\right)}_{e}\right]}^{2}+\sum_{k=1}^{\left|B_{i^{\prime }}\right|}{\left[{\left(x_{{i^{\prime }}_{k}}\right)}_{e}-{\left({\bar{x}}_{i^{\prime }}\right)}_{e}\right]}^{2}\right]/\left(\left|M_{i^{\prime }}\right|+\left|B_{i^{\prime }}\right|-2\right)}$$ (7)

where ${\left(y_{{i^{\prime }}_{k}}\right)}_{e}$ and ${\left(x_{{i^{\prime }}_{k}}\right)}_{e}$ are the *e*-th element of vectors $y_{{i^{\prime }}_{k}}$ and $x_{{i^{\prime }}_{k}}$, respectively, ${\left({\bar{y}}_{i^{\prime }}\right)}_{e}=\sum_{k=1}^{\left|M_{i^{\prime }}\right|}{\left(y_{{i^{\prime }}_{k}}\right)}_{e}/|M_{i^{\prime }}|,\;{\left({\bar{x}}_{i^{\prime }}\right)}_{e}=\sum_{k=1}^{\left|B_{i^{\prime }}\right|}{\left(x_{{i^{\prime }}_{k}}\right)}_{e}/|B_{i^{\prime }}|$, and ${{(\bar{\bar{m}}}_{i^{\prime }})}_{e}=(|M_{i^{\prime }}|{\left({\bar{y}}_{i^{\prime }}\right)}_{e}+{\left|B_{i^{\prime }}|({\bar{x}}_{i^{\prime }}\right)}_{e})/(|M_{i^{\prime }}|+|B_{i^{\prime }}|)$. The test statistic (*T*_*i*′_)_*e*_ measures the distance between the functional connectivity of males and females of group *i’* on the specific link *e*. To measure the global distance between males and females over all links of the set *L*, we can then compute the sum statistic (*T*_*i*′_)_*e*_ over all links:

$$T_{i^{\prime }}=\sum_{e\in E}{\left(T_{i^{\prime }}\right)}_{e}$$ (8)

For testing (6) we can now perform a permutation test based on statistic (8). Specifically, we compute the test statistic (8) over all possible permutations of data across the units. The *p*-value of the test is the proportion of permutation leading to a permuted test statistic higher than the test statistic observed on the non-permuted data.

Let us define as *p*_*i*′_ the *p*-value of the permutation test of (6) for comparing males and females of group *i’*. Now, note that we are performing in this analysis seven different tests of comparison between males and females over the seven groups of age. As a consequence, the results of the seven tests have to be adjusted to take into account the multiplicity [43]. This can be done in several different ways, depending on the type of error that has to be controlled over the family of tests. In this work, we propose to control the family-wise error rate (FWER) by means of the Bonferroni-Holm procedure [44], [45]. As a result, the *p*-values *p*_*i*′_ are adjusted for multiplicity, providing a family of adjusted *p*-values ${{\{\tilde{p}}_{i^{\prime }}\}}_{i^{\prime }=1,\ldots ,7}$. The groups presenting significant differences between males and females can then be selected as the ones with corresponding adjusted *p*-value ${\tilde{p}}_{i^{\prime }}<5\%$. Such a selection is provided with a strong control of the FWER. Specifically, the probability of selecting at least one false positive group (i.e., an age group *i’* with no differences between males and females but an adjusted *p*-value ${\tilde{p}}_{i^{\prime }}<5\%$) is lower than 5%.

### Local analysis

The test (6) performed in the global analysis involves the whole distribution of the *Q*-dimensional random vectors ***Y***_*i*′_ and ***X***_*i*′_, resulting in a single adjusted *p*-value for each age group. If we have enough evidence to state that there is a significant difference between males and females for the age group *i’*, it is then of great importance to identify the specific links that present a significant difference between males and females. This is the scope of the subsequent local analysis, described in this section.

Assume that–for the group *i’*–the adjusted *p*-value is ${\tilde{p}}_{i^{\prime }}<5\%$. For all links *e* ∈ *L* we now aim at testing the null hypothesis of equality between the distribution of (***Y***_*i*′_)_*e*_ and (***X***_*i*′_)_*e*_ against the alternative hypothesis of a difference between the two distributions:

$$H_{0_{i\prime }}:{\left(Y_{i^{\prime }}\right)}_{e}={\left(X_{i^{\prime }}\right)}_{e}\;\mathrm{against}\;H_{1_{i^{\prime }}}:{\left(Y_{i^{\prime }}\right)}_{e}\neq {\left(X_{i^{\prime }}\right)}_{e}.$$ (9)

Note that, with respect to test (6), what now changes, is the fact that now we are performing a univariate test on each single link *e*.

Consistently with the global analysis, the tests (9) can be performed by means of non-parametric permutation tests, by computing the test statistic (7) over all possible permutations of data across the units. The *p*-value of the test is the proportion of permutation leading to a permuted test statistic higher than the test statistic observed on the non-permuted data.

Let us define as *p*_*i*′*e*_ the *p*-value of the permutation test of (9) for comparing males and females of group *i’* and link *e*. Again, for each group *i’* we are performing *Q* = 29 different tests, each one regarding a specific link. Hence, we need to adjust the obtained *p*-values taking into account multiplicity. We now apply the Bonferroni-Holm method to the family of *p*-values {*p*_*i*′*e*_}_*e*∈*L*_. The corresponding adjusted p-values ${\tilde{p}}_{i^{\prime }e}$ are the final result of the local analysis. Again, by selecting the links with an adjusted *p*-value lower than the desired level, we obtain a selection of significantly different links with a strong control of the FWER.

### Discussion on the statistical results

All results of global and local analyses are shown in Table 10. The table reports–for each age group–the unadjusted *p*-values (second column) and the adjusted p-values (third column). The groups presenting significant differences between males (i.e., the ones with an associated adjusted *p*-value lower than 5%) and females are highlighted in bold. In detail, we found significant differences in group 45–60 (p-value 0.042) and 71–79 (p-value 0.035).

**Table 10:** 

Groups	p-value	Adjusted p-value	Significant Links	Anatomical areas
6–15	0.763	1.000		
16–21	0.026	0.130		
26–35	0.823	1.000		
36–40	0.071	0.284		
**46–50**	**0.007**	**0.042**	**24–36**	Left Intracalcarine Cortex- Left Lingual Gyrus
58–70	0.696	1.000		
**71–79**	**0.005**	**0.035**	**31–78** **(22–31)**	Left Precuneous Cortex-Right Precuneous Cortex (Left Lateral Occipital Cortex-Left Precuneous Cortex)

Results of the global and local analyses: unadjusted and adjusted *p*-values for each age group and significant links. Groups with adjusted *p*-values lower than 5% are highlighted in bold. The link in parentheses is characterized by a *p-*value lower than 10%.

The fourth column of the table reports the significant links emerging from the local analysis on groups with an associated global adjusted *p*-value lower than 5%.

All results of the local analysis for the groups with a global adjusted *p*-value lower than 5% are shown in Table 11. The table reports–for each link–the *p*-value of test (9) and the Bonferroni-holm adjusted *p*-value. The links with local adjusted *p*-values lower than 10% are highlighted in bold.

**Table 11:** 

Group 46–50				Group 71–79			
Link		p-values	adjusted p-values	Link		p values	adjusted p values
**24**	**36**	**0.001**	**0.03**	**31**	**78**	**0.001**	**0.03**
30	77	0.008	0.232	**22**	**31**	**0.003**	**0.087**
69	78	0.009	0.252	28	75	0.006	0.168
31	78	0.012	0.324	22	78	0.01	0.27
28	29	0.012	0.324	28	29	0.014	0.364
29	76	0.013	0.325	30	77	0.018	0.45
30	31	0.024	0.576	30	78	0.018	0.45
28	76	0.026	0.598	75	76	0.028	0.644
75	76	0.031	0.682	29	76	0.03	0.66
31	77	0.036	0.756	69	78	0.031	0.66
28	75	0.078	1	77	78	0.031	0.66
29	75	0.08	1	48	75	0.039	0.741
77	78	0.083	1	28	48	0.041	0.741
31	69	0.084	1	21	22	0.046	0.782
48	75	0.097	1	47	94	0.067	1
22	69	0.124	1	24	36	0.082	1
47	94	0.133	1	30	31	0.088	1
36	83	0.191	1	22	69	0.09	1
30	78	0.204	1	31	77	0.091	1
22	31	0.237	1	1	48	0.102	1
22	78	0.355	1	28	76	0.103	1
69	70	0.486	1	1	28	0.167	1
22	77	0.566	1	23	47	0.205	1
1	48	0.601	1	36	83	0.305	1
23	47	0.601	1	69	70	0.728	1
1	28	0.635	1	29	75	0.776	1
28	48	0.714	1	31	69	0.803	1
21	22	0.929	1	54	64	1	1
54	64	1	1	22	77	1	1

Results of the local analysis for groups 46–50 and 71–79: for each link, the table reports the *p*-value and the adjusted *p*-value. The links with associated adjusted *p*-values lower than 5% are highlighted in red and bold, while the ones with associated adjusted *p*-values lower than 10% are highlighted in blue and bold.

We found out one significant link in the age class 46–50, that is the link 24–36 with associated adjusted p-value equal to 0.03. In the age class 71–19 we found out the significant link 31–78 with associated p-value equal to 0.03. In addition, in the latter class the adjusted p-value of link 22–31 is equal to 0.087, suggesting a weak evidence that also this link is different between males and females.

Note that the sample size in all age classes is not very high. The use of permutation tests, in this case, is of capital importance since they enable to obtain exact tests for every sample size. The statistical power of the inferential analysis might, however, be low due to the low sample sizes. However, it is important to note that, despite a possibly low power, it is possible to obtain significant results. This suggests that the significant results reported in the paper are valid, and potentially they could be improved (for instance, the number of significant links could be increased) by increasing the sample size.

## Comparison of models

The FD model [20] needs, for the calculation of the intensity of functional connectivity between couples on nodes (representing cerebral areas), not only the functional data but also the degree of such nodes and their distance. We point out again that, generally, only the functional data are used in the literature.

Therefore, we are naturally invited to investigate analogies and differences between the usual approach which employs statistical correlations (we remind the reader we shortly called pFC approach and pFC data, where pFC stands for pure functional connectivity), and the results obtained with the adopted FD model.

Such a comparison could be carried out with different procedures. Here we decided to follow a smooth approach, meaning that we considered the average functional connectivity of all subjects.

To this, we compacted the 133 matrices ***W***_***k***_, *k* ∈ {1,…,94}, into a single matrix ***W***^***rep***^, of size 94x94, whose entry ***W***^*rep*^(*i*,*j*) is the average value of the 133 entries at position *(i*,*j)* in the matrices ***W***_***1***_, ***W***_***2***_,*…*,***W***_***133***_. In other words, $W^{rep}(i,j)=\frac{W_{1}(i,j)+W_{2}(i,j)+\cdots +W_{133}(i,j)}{133}$, where *i*,*j* ∈ {1,…,94}.

Note that, now, we do not separate the contribution of males from that of females.

Analogously, we compacted the 133 pure functional connectivity matrices **F**_**k**_, *k* ∈ {1,…,94}, to get a single matrix ***F***^***rep***^, still of size 94x94, where $F^{rep}(i,j)=\frac{F_{1}(i,j)+F_{2}(i,j)+\cdots +F_{133}(i,j)}{133}$, *i*,*j* ∈ {1,…,94}.

From ***W***^***rep***^ and ***F***^***rep***^ we formed the matrix ***M*** *=* ***W***^***rep***^***—F***^***rep***^. Then we selected only the entries of ***M*** greater than 0.1116, and setting to zero all the remaining ones. Such a threshold has been derived from the analysis of the histogram of the distribution of the entries of ***M***, which shows a clear separation between two different sets. The resulting number of selected entries is four, so it is very limited, which points out that both models find almost the same links at resting state. Such four links are:

- 31–78 (left precuneus–right precuneus)
- 59–89 (right middle temporal gyrus, posterior division–right central opercular cortex)
- 59–93 (right middle temporal gyrus, posterior division–right planum temporal, posterior division)
- 12–42 (left middle temporal gyrus, posterior division–left central opercular cortex)

We are induced to ascribe functional importance to the above connections, even if the corresponding nodes do not directly belong to the DMN. Actually, we cannot exclude that they could play a role of some importance at resting state since they could be related to other resting state functional networks. Indeed, the precuneus is well known to be a very interconnected area [46]. Moreover, the connection between the precuneus and the DMN at resting state (as well as during specific tasks) is justified by some studies in literature (see for example [47]).

Regarding the links 59–89 (*middle temporal gyrus*, *posterior division right-central opercular cortex*, *right*) and 59–93 (*middle temporal gyrus*, *right-planum temporal posterior division*, *right*), they are anatomically and reciprocally connected [48]. Despite the role of link 12–42 (*left middle temporal gyrus*, *posterior division*, *left central opercular cortex*) is not totally clear, it seems that the posterior part of the middle temporal gyrus could be functionally related to the DMN. In fact, it is recruited in automatic semantic processing [49], which is not so surprising during a resting state condition.

The above speculations should be intended as a preliminary indication in view of possible future analysis aiming at clarifying the precise role of the involved brain regions at resting state.

## Conclusions

In this paper we have investigated the FC intensity changes, and some related invariant properties, in healthy people across the life span and at resting state. To this, we have exploited the recently proposed FD model [20], and we have applied it to a sample of 133 healthy participants, with age distributed throughout the lifespan. The main novelty of this approach, compared to those typically used, includes the modification of the way of weighting the brain network edges, which is done by accounting for the node degree and the physical distance between nodes, as well as the correlation between the fMRI time course.

After grouping the 133 subjects into 28 different groups (14 of males and 14 of females) according to their age, we used the FD model and the related thresholding procedure to identify the strongest links within each one of the groups. This leads to the construction of a representative graph *G(V*,*E)* of the neural network over the life span and at resting state.

In order to investigate the differences in connectivity between males and females in the different age classes, we have performed a careful statistical analysis based on the set *L* of emerging links. We found out one significant difference in the age classes 46–50 and 71–19, that we have extensively commented. In addition, in the latter class the adjusted p-value of link 22–31 is equal to 0.087, suggesting a weak evidence that also this link is different between males and females.

Analogies and differences have been also investigated between the usual approach, i.e. the analysis led with the pFC data, and the results obtained with the adopted FD model. We found that all the most important link at resting state, found via the pFC analysis, are found also with the FD model, which remarkably contributes to the validation of the proposed model. Furthermore, by means of the FD model analysis we found three further links, not selected by the pFC analysis. The corresponding nodes do not directly belong to the DMN but could connect to other resting state functional networks. This would deserve a specific topic for future studies.

The global analysis shows that there are not great differences, in terms of functional connectivity intensity, between healthy males and females at resting state. Indeed, only two age groups present significant differences in functional connectivity between males and females. The two groups are subjects from 46 to 50 years (adjusted p-value ${\tilde{p}}_{i^{\prime }}=0.042$)) and subjects from 71 to 79 years (adjusted p-value ${\tilde{p}}_{i^{\prime }}=0.035$). All other groups have adjusted p-values higher than 10%, meaning that they show significant analogies in functional connectivity between males and females. Note that similar conclusions can be drawn from the unadjusted p-values, meaning that in this case, the multiplicity adjustment is not highly conservative.

The local analysis performed on the two groups showing significant differences enables us to select at least one link showing significant differences between males and females. Interestingly, such link is not the same in the two age groups emerging from the global analysis: we select at 5% level the link 24–36 for the group 46 to 50 years, and the link 31–78 for the age group 71 to 79 years. In addition, for this latter group, the link 22–31 has an adjusted p-value lower than 10%. In this case, note that the number of tests that are jointly performed is higher than in precedence (29 against 7). As a consequence, the adjustment for multiplicity is also stronger. While there is a lot of links with associated unadjusted p-values lower than 5%, the ones with adjusted p-values lower than such threshold reduce to only one for each group. This latter consideration suggests that the adjustment performed by the Bonferroni-Holm method might be too conservative in this case, and suggests performing a deeper analysis (e.g., based on a higher number of subjects) to further investigate the differences between the links). This is also why we suggest using a higher value of FWER (i.e., 10%) for selecting links in this case.

Note that–being all analyses based on non-parametric permutation tests–we do not need to require any parametric model for the data distribution. In particular, we do not assume that data are distributed as a Gaussian, and even in presence of non-Gaussian data, the results reported here are exact. The only assumption that is needed here is that the data belonging to the same group are independent and identically distributed.

Regarding the model comparison analysis, the adopted strategy pointed out a few connections that seem to have an active role at resting state. Even if the corresponding nodes are not always directly related to the DMN, they should be considered in view of possible deeper analysis.

Our results are intended to be a contribution in shedding more light on the neuroscience of the resting state. This topic is fundamental in translational neuroscience, just think about the role of the (different) development of functional and structural connectivity in males and females [50], [51]. The DMN is associated with the free wandering of the human mind, one’s self, i.e. one’s reminiscence of the past, one’s introspection of the present thoughts, feelings and one’s plans for the future [52], this wandering could reflect the inner mental state of the subjects and the basic traits of their personality. Future perspective leads towards the study of DMN and functional connectivity changing over life in males and females affected by neuropsychiatric diseases, as well as the inclusion of possible negative correlations in the model.
